# Comparison of Lysis and Amplification Methodologies for Optimal 16S rRNA Gene Profiling for Human and Mouse Microbiome Studies

**DOI:** 10.3390/ijms26031180

**Published:** 2025-01-29

**Authors:** Farzaneh Rastegari, Mark Driscoll, Jesse D. Riordan, Joseph H. Nadeau, Jethro S. Johnson, George M. Weinstock

**Affiliations:** 1 Department of Computer Science and Engineering, University of Connecticut, 371 Fairfield Way, Storrs, CT 06269, USA; farzaneh.rastegari@uconn.edu; 2 The Jackson Laboratory for Genomic Medicine, 10 Discovery Drive, Farmington, CT 06032, USA; 3 Intus Biosciences, 400 Farmington Ave, Farmington, CT 06032, USA; mark.driscoll@intusbio.com; 4 Department of Anatomy & Cell Biology, Carver College of Medicine, The University of Iowa, Iowa City, IA 52242, USA; riordanj@uiowa.edu; 5 Pacific Northwest Research Institute, 720 Broadway, Seattle, WA 98122, USA; joseph.nadeau@mainehealth.org; 6 Center for Molecular Medicine, MaineHealth Institute for Research, Scarborough, ME 04074, USA; 7 The Oxford Centre for Microbiome Studies, Kennedy Institute of Rheumatology, University of Oxford, Roosevelt Drive, Headington, Oxford OX3 7FY, UK; 8 Genetics and Genome Science, University of Connecticut Health Center, 400 Farmington Ave, Farmington, CT 06030, USA

**Keywords:** DNA extraction, 16S rRNA gene sequencing, microbiome, alkaline-based lysis, bead-beating lysis, V1–V3 16S rRNA gene primers

## Abstract

When conducting sequence-based analysis of microbiome samples, it is important to accurately represent the bacterial communities present. The aim of this study was to compare two commercially available DNA isolation and PCR amplification approaches to determine their impact on the taxonomic composition of microbiome samples following 16S rRNA gene sequencing. A well-established 16S rRNA gene profiling approach, which was widely used in the Human Microbiome Project (HMP), was compared with a novel alkaline degenerative technique that utilizes alkaline cell lysis in combination with a degenerate pool of primers for nucleic acid extraction and PCR amplification. When comparing these different approaches for the microbiome profiling of human and mouse fecal samples, we found that the alkaline-based method was able to detect greater taxonomic diversity. An in silico analysis of predicted primer binding against a curated 16S rRNA gene reference database further suggested that this novel approach had the potential to reduce population bias found with traditional methods, thereby offering opportunities for improved microbial community profiling.

## 1. Introduction

High-throughput sequencing technologies have advanced microbiome research by enabling the study of diverse and complex microbial communities. At the core of this research lies the need for efficient DNA extraction that maintains DNA integrity while ensuring the faithful and reproducible representation of bacterial diversity. Overcoming the challenge of lysing diverse bacterial cell walls without compromising DNA quality therefore represents a critical step in sequence-based microbiome studies.

Existing methods of DNA extraction can introduce biases due to their differing physical, chemical, and enzymatic approaches. A critical factor influencing these biases is the cell wall composition of the target bacteria. One example is bead-beating, a mechanical disruption method commonly used to break open bacterial cells. It utilizes high-speed agitation with small beads to physically disrupt cell walls. While effective for many bacteria, bead-beating can be challenging for Gram-positive bacteria due to their thick peptidoglycan layer. The rigidity of this layer can hinder the beads from fully lysing Gram-positive cells, leading to a reduced DNA yield (Figueroa-Bossi, 2022 [1]). By contrast, enzymatic lysis may be less effective against Gram-negative bacteria. This can be attributed, in part, to the presence of an outer lipopolysaccharide (LPS) membrane, which enzymatic treatments might have difficulty penetrating. LPS can also disrupt the effectiveness of detergents commonly used in lysis buffers, further hindering the enzymatic degradation of the cell wall (Koshy et al., 2017 [2]). Furthermore, overly harsh reaction conditions, such as excessive bead-beating, can damage extracted DNA, making it unsuitable for downstream analyses like metagenomic sequencing (Corcoll et al., 2017 [3] and Wesolowska-Andersen et al., 2014 [4]).

The use of sodium hydroxide (NaOH) for DNA extraction has been proposed as an alternative to enzyme and tissue disruption-based approaches for DNA extraction. However, in addition to aiding in the breakdown of the cell wall, NaOH also breaks down the hydrogen bonds that bind the bases of DNA, resulting in the conversion of all double-stranded DNA, including the plasmid and genomic DNA (gDNA), to single-stranded DNA, making it less favorable for applications requiring high-quality genetic material (Birnboim and Doly in 1979 [5]).

Following DNA extraction, 16S ribosomal RNA gene amplification and sequencing is one of the most widely used and cost-effective methods for quantifying the relative abundance of different bacteria in microbiome samples (Gibbons et al., 2015 [6]). It has the added advantage of being compatible with single-strand DNA templates, therefore overcoming one potential limitation of alkaline-based extraction methodologies.

Various approaches have been proposed to target specific variable regions within the 16S ribosomal gene, with most employing a single primer pair to perform PCR amplification prior to sequencing. Use of restricted primer sets can, however, introduce additional biases due to sequence mismatches at the primer annealing sites within the 16S rRNA gene sequence. These mismatches can lead to thermodynamic instability between the primer and target DNA, resulting in decreased primer–template annealing strength. Consequently, the efficiency and specificity of PCR amplification can be negatively affected. This translates to an underrepresentation or complete exclusion of certain bacterial taxa in the final sequencing data (Klindworth et al., 2013 [7]). For example, *Bifidobacterium*, a genus of significant interest in human gut microbiome research (Fujiyoshi et al., 2020 [8]), is a taxon potentially underrepresented due to mismatches with the commonly used 27F primer. Studies by Graf et al. (2021 [9]) have shown that many *Bifidobacterium* species exhibit mismatches with the forward primers used by the Human Microbiome Project (HMP), potentially leading to a significant underestimation of *Bifidobacterium* abundance in the final sequencing data (Klindworth et al., 2013 [7]).

Recently, Intus Biosciences introduced a novel, rapid alkaline-degenerative method for microbial DNA extraction and profiling (Hong et al. [10]). This approach uses potassium hydroxide (KOH) to effectively degrade both Gram-positive and Gram-negative cell walls. In addition, it employs multiple degenerate 16S gene primers during PCR amplification, increasing the likelihood of binding with minimal or no mismatches between the primer and 16S gene during PCR.

This study aimed to extend the work of Hong et al. to compare this novel approach to the more established method for DNA extraction and 16S rRNA gene amplification that was widely used by the HMP [11]. Henceforth, we will refer to these alternative approaches as ‘Rapid’ and ‘HMP’, respectively. Extending the work of Hong et al., we compared their efficacy for the analysis of fecal samples collected from both humans and mice. We further performed an in silico analysis to evaluate whether differences in observed microbiome composition between these two approaches may result from the different cell lysis approaches or the use of degenerate PCR primer sets. Collectively, these two studies sought to refine DNA extraction methodologies for microbiome profiling by optimizing the 16S rRNA gene extraction method and enhancing the diversity of 16S rRNA sequences that can be efficiently amplified in downstream PCR amplification.

## 2. Results

### 2.1. The Intus Rapid Technique Detects Greater Diversity in Fecal Samples than Established HMP Methods

**Figure 1:**
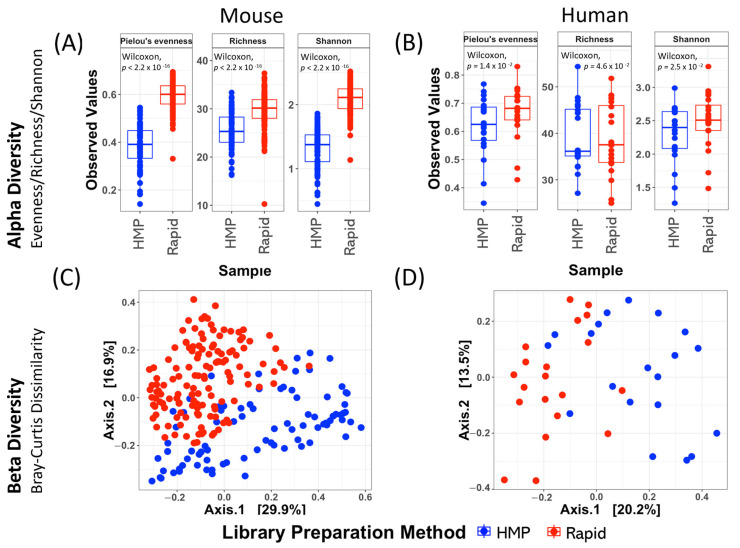
Method effects on the evidence for microbiome diversity and sample similarity: a comparison of HMP vs. Rapid. (**A**,**B**) Alpha diversity calculated at the genus level for mouse and human stool samples extracted using either the HMP or Rapid technique. Each data point represents the alpha diversity measurement for one sample. Boxes show the distribution of data points within each group, with a line showing the mean value. Wilcoxon *p*-values for the mouse dataset and pairwise Wilcoxon *p*-values for the human dataset are shown on each panel. (**C**,**D**) PCoA plots to represent the difference in microbiome community composition between samples. Each data point represents a single sample. The percentage values on each axis represent the proportion of variance explained by each of the first two principal coordinate axes. ANOSIM test results confirmed significant differences in the microbial community composition between groups: mouse dataset (R = 4.32 × 10^−1^, *p* < 10^−3^) and human dataset (R = 2.898 × 10^−1^, *p* < 10^−3^).

Community-level comparisons of microbiomes extracted using different approaches revealed that the Rapid approach detected a greater diversity of bacterial genera in mouse feces, as indicated by multiple alpha diversity measures (Figure 1A). The same trend was apparent for human feces and was statistically significant in a reanalysis of the data of Hong et al. [10] (Figure 1B). Principal coordinate analysis of beta diversity based on Bray–Curtis distance matrices also indicated that the different DNA extraction/PCR amplification methods exerted a significant effect on microbiome community composition measured in both mouse (Figure 1C, ANOSIM R = 4.32 × 10^−1^, *p* = 10^−3^) and human samples (Figure 1D, ANOSM R = 2.90 × 10^−1^, *p* = 10^−3^). While results reported in the main text were based on an analysis of the diversity of bacterial genera, the same trends were observed when analyzing diversity at the level of operational taxonomic units (OTUs, Supplementary Figure S1). The extraction and PCR amplification approach also exerted a greater effect on microbiome composition than other potential sources of variation, such as mouse strain or the health status of human patients (Supplementary Figure S2).

### 2.2. Different 16S rDNA Preparation Methods Are Biased Towards Different Organisms in Mice and Humans

**Figure 2:**
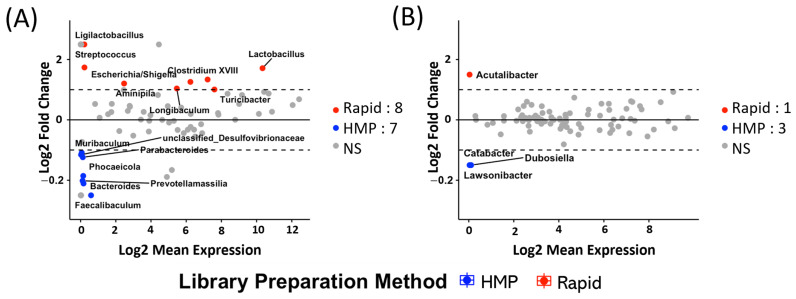
Microbiome variations between 16S rDNA extraction methods (HMP vs. Rapid) using fold change analysis. MA plot for the log2(fold change) of organisms in samples of the mice fecal (**A**) and human stool (**B**) datasets. MA plots were utilized to illustrate the log2(overall mean abundance percentage) of all organisms on the x-axis and the log2(fold change) between the mean abundance percentage of the organisms found in samples processed by Rapid and HMP lysing procedures on the y-axis. Dotted lines show the cutoff log2(fold change) values (log2(fold change) = −1 and log2(fold change) = 1). The *p*-value for each estimated log2(fold change) of each genus was determined with a *t*-test (refer to Supplemental MicrobiomeData_PrimerSets_StatisticalResults.xlsx), and the significant genera were adjusted for a *p*-value threshold of <0.05.

Community-level differences in microbiome composition were followed up with analysis to identify individual taxa whose estimated relative abundance was altered as a consequence of the preparation method. Preliminary exploration *t*-tests were applied, and a log2(fold change) threshold (−1 < log2(fold change) < 1) was set to identify organisms overrepresented by one or another approach based on their mean relative abundance. In mouse fecal samples (Figure 2A), eight genera were significantly enriched as a consequence of applying the Rapid approach, while seven genera were significantly enriched as a consequence of applying the HMP approach. Notable genera enriched as a consequence of applying the Rapid approach included *Lactobacillus*, *Clostridium XVIII*, *Escherichia/Shingella*, *Aminipila*, *Streptococcus*, and *Ligilactobacillus*. Genera enriched as a consequence of applying the HMP approach were *Faecalibacterium*, *Bactroides*, *Phocaeicola*, *Parabacteroides*, *Prevotellamassilia*, *Muribaculum,* and *unclassified_Desulfovibrionaceae*. In human samples, the Rapid approach resulted in the significant enrichment of the genus *Acutalibacter*, while the HMP approach resulted in the significant enrichment of the genera *Dubosiella*, *Catabacter,* and *Lawsonibacter* (Figure 2B). A greater number of genera were differentially abundant in mice fecal samples than in human stool samples, which may be due to the limited number of human stool samples available for analysis.

### 2.3. A Combined Significance Ranking Score Identifies the Most Consistent Genera That Discriminate Between Rapid and HMP Protocols

Multiple approaches exist to identify the taxa driving the differences in microbiome composition. To mitigate potential methodological bias associated with any one approach, we applied seven different statistical and machine learning methods to identify taxa that distinguished between the Rapid and HMP samples. By combining significance scores from these analyses, we determined the top 20 genera most consistently differentially abundant between the two sample processing methods (Table 1). The complete list of organisms detected in our datasets is provided in Supplementary Material (MicrobiomeData_PrimerSets_StatisticalResults.xlsx).

### 2.4. Rapid V1–V3 Primers Contain Fewer Mismatches to Binding Sites in 16S rRNA Genes of Reference Taxa

While differences in the relative abundance of individual taxa observed between the two methods may be attributable to extraction approaches, they may also be due to the use of degenerate primers as part of the Rapid protocol, which may result in greater binding affinity in taxa with mismatches to the original 27F/357FR primer combination used in the HMP protocol. To determine whether biases in the most differentially abundant taxa were due to the extraction technique or primer bias, we examined the primer specificity for the top 20 differentially abundant genera identified in Table 1. Comparing primer sequences to a custom database of 12,990 curated, taxonomically annotated 16S rRNA gene sequences (Athena Database, see methods), for each of the top 20 genera, we determined the number of primers providing an exact match to one or more reference sequences assigned to that genus (Table 1, column 2). We further recorded the number of OTUs detected by each method in our data (Column 3) and the prevalence of each genus in our data (Column 4). By comparing these parameters, we aimed to elucidate the relative contributions of extraction versus primer bias to the observed differences in taxa abundance between the Rapid and HMP methods.

**Figure 3:**
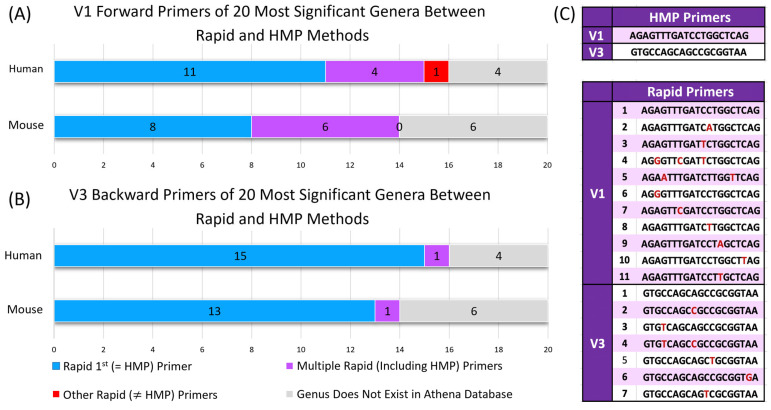
Primer coverage for the V1–V3 region was assessed across the 20 most consistent genera that discriminated between the Rapid and HMP methods in human and mouse microbiome studies. The analysis focused on V1–V3 primers and their availability in the Athena database, which curates 16S rDNA gene reference sequences. (**A**) V1 forward primer: humans: At least one primer (HMP or Rapid) was available for 15 out of 16 genera in the Athena database. Four genera had multiple Rapid primers, and one genus (*Collinsella*) had a primer with a single mismatch to the HMP primer. Mice: All 14 genera in the Athena database had at least one primer (HMP or Rapid). Six genera had multiple Rapid primers. (**B**) V3 reverse primer: humans: At least one primer (HMP or Rapid) was available for all 16 genera in the Athena database. One genus (*Bacteroides*) had multiple Rapid primers. Mice: At least one primer (HMP or Rapid) was available for all 14 genera in the Athena database. One genus (*unclassified_Firmicutes*) had multiple Rapid primers. (**C**) List of V1 forward and V3 reverse primers for the HMP and Rapid techniques. The red letters in the Rapid primer set indicate at that base position compared to the corresponding HMP primer.

Of the 20 mouse genera that consistently distinguished between Rapid and HMP, 14 were represented by at least one reference sequence in the Athena database, and six of these 14 genera had multiple matches within the Rapid forward/reverse degenerate primer sets (Figure 3A): *Lactobacillus* (primers 1 and 9), *unclassified_Lachnospiraceae* (primers 1 and 7), *unclassified_Clostridiales* (primers 1, 6, 7, and 9), *unclassified_Bacteroidales* (primers 1 and 6), *unclassified_Firmicutes* (primers 1, 6, 7, 8, and 9), and *unclassified_Ruminococcaceae* (primers 1 and 6). Of the 20 human genera that consistently distinguished between the Rapid and HMP methods, 16 were represented by at least one reference sequence in the Athena database, and 4 genera had multiple matches within the Rapid forward/reverse degenerate primer sets (Figure 3B): *Bacteroides* (primers 1 and 9), *unclassified_Ruminococcaceae* (primers 1 and 6), *Collinsella* (primer 7), *unclassified_Lachnospiraceae* (primers 1 and 7), and *unclassified_Clostridiales* (primers 1, 6, 7, and 9).

When considering genera that consistently distinguished between Rapid and HMP in mice and humans, a single genus (*Collinsella*) represented in the Athena database failed to match the HMP forward primer. This genus had an exact match to a single forward primer within the Rapid degenerate primer set. When this analysis was extended to all genera found in the samples (Supplemental MicrobiomeData_PrimerSets_StatisticalResults.xlsx), two organisms in the mice dataset and three in the human dataset failed to match the HMP primers. Notably, this included the genus *Bifidobacterium*, which had three mismatches within the HMP forward primer; however, the Rapid primer set contained two primers with exact matches to the representative *Bifidobacterium* sequences in the Athena database.

The number of OTUs assigned to each genus is also presented in Table 1. Interestingly, for multiple genera, the number of OTUs detected varied between methods. For example, using the Rapid approach, two OTUs of *Lactobacillus* (the most discriminatory genus in the mouse dataset) were identified, but only one OTU was discovered using the HMP method. By contrast, using the HMP approach, twelve OTUs were found for *Bacteroides* (the most significant genus in the human dataset), but the Rapid method only detected seven. The number of OTUs detected for a given genus also appeared to broadly correlate with the prevalence with which that genus was detected across samples.

## 3. Discussion

Microbiome profiling using 16S rRNA gene sequencing can suffer from technical biases. Among these are the potential for different DNA extraction methods to either fail to effectively lyse bacterial cells or to over-process cells, leading to incomplete and damaged DNA prior to PCR amplification. Variations in bacterial cell wall structures mean these biases can differentially affect specific taxa, leading to biases in the estimates of microbiome community composition (Carrigg et al., 2007 [12] and Krsek et al., 1999 [13]). Additionally, natural variation in conserved regions of the 16S gene used for PCR primer binding can result in further biases, with some taxa failing to amplify effectively due to mismatches with restricted primer sets (Abellan-Schneyder et al., 2021 [14]). This study investigated the influence of two different extraction and PCR amplification approaches on microbiome profiling accuracy. We compared the well-established HMP (bead-beating) method to the novel Rapid method (alkaline-based lysis) recently introduced by Intus Biosciences [10].

Notably, the utilization of these two methods resulted in statistically significant differences in estimates of microbiome composition (beta diversity), and the Rapid method resulted in significantly higher alpha diversity when applied to fecal microbiome samples collected from both humans and mice. While it is not possible to ascertain the true composition of ex vivo microbiome samples, greater alpha diversity—in particular, taxonomic richness—suggests the Rapid approach is capable of detecting a greater number of bacterial taxa in samples than the HMP approach. This is consistent with previous reports that alkaline-based nucleic acid extraction approaches are capable of lysing a wide diversity of bacterial cell types (Shwani et al., 2024 [15]).

Greater evenness further suggests that the Rapid approach may result in better representation of the relative abundance of taxa present in a sample. While OTUs are an imperfect approximation of taxonomic diversity (Mysara et al., 2017 [16]), a greater number of OTUs assigned to the same genus may reflect greater diversity at the species or strain level. Where such diversity exists within a genus, it is likely that degenerate primers would be better able to detect it. The Rapid approach frequently detected a greater number of OTUs assigned to murine genera, whose relative abundance consistently discriminated between methods. However, this same trend was again not consistent for human genera, where neither method consistently detected more OTUs.

Further evidence that the inclusion of degenerate primers may lead to improved taxonomic representation is the failure of the HMP primers to generate an exact match for binding sites in sequences representing key genera (e.g., *Collinsella*, *Bifidobacteria*) present within our reference database. This did not preclude the detection of these genera within actual samples, suggesting that reduced annealing efficiency is still sufficient for detection. It is, however, consistent with our observation that these taxa were detected at a reduced presence and relative abundance, as well as with previous studies that reported limitations in the detection of these taxa with conventional primer sets (Graspeuntner et al., 2018 [17], Matsuo et al., 2021 [18], Kim et al., 2013 [19], and Kai et al., 2019 [20]).

One limitation of our current study is that it is unable to fully separate the influence of primer bias versus DNA extraction methods on microbiome composition. While this could be achieved by combining conventional and degenerate primer sets with both the HMP and Rapid extraction approaches, our current study utilized only commercially available kits and their associated methodologies. Accepting this limitation, our in silico analysis strongly suggests that primer selection is at least in part responsible for any improvements in microbiome representation achieved by the Rapid kits. Thus, while future work could definitively quantify the relative extent to which the lysis method and primer choice impact the estimates of microbiome composition, here, we showed that both are likely to be important and are likely to contribute to the greater diversity estimates observed with Intus kits.

A second limitation of this study is its focus on a single variable region (V1–V3). Multiple variable regions (e.g., V4, V3–V4) were targeted in 16S studies using short-read technologies, whereas the full 16S rRNA gene (incorporating variable regions V1–V9) can be sequenced using long-read technologies. Such variety in 16S approaches means that specific taxonomic biases reported here may not be directly applicable to studies targeting other regions of the 16S rRNA gene. In spite of this, the greater sensitivity reported for the Rapid approach is likely to transcend different variable regions.

In conclusion, we provided a detailed comparison of a novel method for alkaline-based DNA extraction and 16S rRNA gene amplification against a well-established conventional approach. In doing so, we demonstrated that the Intus Rapid approach has a significant impact on measurements of the microbiome community composition, likely driven by greater sensitivity attributable to a combination of cell lysis methodology and primer selection. This further highlights the fact that through careful selection of appropriate DNA extraction and PCR amplification methodologies, researchers can enhance the reliability and generalizability of their microbiome research findings.

## 4. Materials and Methods

This study compared the effectiveness of two methods for the extraction and PCR amplification of 16S rDNA gene sequences from human and mouse samples, the first being a commercially available protocol (Qiagen PowerSoil kit, manufactured by Qiagen, Hilden, Germany) used extensively in HMP and the second being a novel ‘Rapid’ KOH alkaline-based protocol recently introduced by Hong et al. [10].

### 4.1. Sample Collection

#### 4.1.1. Mouse Fecal Microbiome Samples

Fecal samples were collected from a total of 220 mice comprising 22 distinct strains (Nadeau et al., 2000 [21] and Singer et al., 2004 [22]). Two of the 22 strains were A/J and C57BL/6, with the remaining 20 strains consisting of a single A/J chromosome substituted onto a C57BL/6 background. Full nomenclatures for each strain can be found in Supplementary Material (MicrobiomeData_PrimerSets_StatisticalResults.xlsx). Mice were bred and maintained at The Jackson Laboratory (Bar Harbor, ME, USA), where they were fed a standard chow diet. Feces were collected when mice were five weeks of age and stored at −80 °C until processing. Of the 220 mouse fecal samples collected, 84 were extracted using the HMP approach, while the remaining 136 were extracted using the Rapid approach.

#### 4.1.2. Human Oral Microbiome Samples

Raw sequencing data from 20 human oral microbiome samples used in the study by Hong et al. [10] were accessed for reanalysis. The original data were from a cancer study that recruited ten hospitalized lung cancer patients and ten healthy controls for stool sample collection, with further details of sample collection provided in [10].

### 4.2. DNA Extraction, 16S rRNA Gene Amplification, and Sequencing

#### 4.2.1. Qiagen PowerSoil Kit Protocol (HMP)

Fecal samples from 84 mice (2 A/J, 2 C57BL/6J, and 4 from each of the other 20 CSSs) were processed using the Qiagen Mo Bio PowerSoil DNA Isolation Kit protocol [23], which reflected methods commonly employed by the Human Microbiome Project. Briefly, fecal samples underwent a two-step thermomechanical lysis. In the initial step, samples were pre-treated at 65 °C for 10 min. This was followed by a heat shock at 95 °C for 10 min. Following pre-treatment, the pre-heated fecal suspension was transferred to PowerBead tubes and combined with 60 μL of lysis solution (C1, sodium dodecyl sulphate). Using a MO BIO Vortex Adapter (manufactured by MO BIO Laboratories, Carlsbad, CA, USA, acquired by Qiagen, Hilden, Germany) tube holder, bacterial cells were subjected to 10 min of bead-beating at 4000 rpm. Cellular debris was then pelleted by centrifugation at 10,000× *g* for 10 min. The resulting DNA-containing supernatant was washed using silica spin columns and purified DNA eluted to a volume of 50 μL. The 27F forward (5′-AGAGTTTGATCCTGGCTCAG-3′) and 357R reverse (GTGCCAGCAGCCGCGGTAA) primers were used for the PCR amplification of the V1–V3 region of the 16S rRNA gene. AccuPrime Taq DNA Polymerase High Fidelity and 10X AccuPrime Buffer II (Invitrogen, Carlsbad, CA, USA, a Thermo Fisher Scientific brand headquartered in Waltham, MA, USA, 12346086) were utilized for amplification, with 30 cycles of denaturation at 94 °C for 15–30 s, annealing at 56 °C for 15–30 s, and extension at 68 °C for 1 min/kb. For PCR-positive controls, 4 ng of a DNA standard consisting of the microbial mock community B (even, low concentration) (BEI Resources, Manassas, VA, USA) was used, while nuclease-free water was utilized for PCR-negative controls. PCR products were purified with Agencourt AMPure XP beads (Beckman Coulter, Brea, CA, USA, A63882) and measured with the Qubit dsDNA HS Kit (Thermo Fisher Scientific, Waltham, MA, USA, Q32854). PCR products were pooled at equimolar concentrations into a single library (4 nM final concentration) using the Illumina Library Quantification Kit (ROX Low qPCR Mix, Illumina, San Diego, CA, USA, KK4873) and the Illumina Library Quantification Standards 1–6 (Illumina, San Diego, CA, USA, KK4903). The quality of the library was evaluated using the 4200 TapeStation System with the High Sensitivity D1000 ScreenTape assay (Agilent Technologies, Santa Clara, CA, USA). Sequencing was performed with a 250 bp paired-end sequencing methodology on the Illumina MiSeq platform.

#### 4.2.2. Intus Biosciences V1–V3-Illumina Kit Protocol (Rapid) [24]

Fecal samples from 136 mice (8 A/J, 8 C57BL/6, and 6 from each of the CSSs) were processed using the novel rapid alkaline approach introduced by Hong et al. [10]. Rapid incorporated a proprietary, chemically based, high-throughput lysis and PCR process. Following the manufacturer’s instructions, first, 1–3 mg of fecal material was collected with a calibrated 10 μL inoculating loop and thoroughly dispersed in 50 μL of lysis buffer (2% by weight sodium dodecyl sulfate) by twisting the loop. To facilitate cell lysis and DNA release, 50 μL of 0.4 M KOH solution was then added, followed by incubation at 95 °C for 10 min in a thermal cycler. The lysate then cooled, allowing a visible pellet to form. The supernatant containing the extracted DNA was carefully transferred to a new tube. Next, 50 μL of purification buffer (sodium chloride solution with magnetic beads) was added to the supernatant, and the mixture was incubated at 50 °C for 5 min to promote DNA binding to capture the beads present in the buffer. To remove impurities, the beads were washed twice with 70% ethanol solution. Finally, purified DNA was eluted from the capture beads using 40 μL of TE buffer. The eluted DNA was diluted further by adding 160 μL of fresh TE buffer, resulting in a final volume of 200 μL. From this diluted solution, 10 μL was used for target gene PCR amplification.

PCR amplification employed a custom pool of barcoded primers consisting of 10 degenerate forward primers in combination with 6 degenerate reverse primers to capture the V1–V3 region (Figure 3). Notably, these primers included the 27F and 357R primer pair used in the HMP method. PCR reactions were performed using a single reaction mixture containing all 10 forward primers and all 6 reverse primers at a final concentration of 0.2 μM each. Amplification and sequencing processes were performed as described for the HMP method.

### 4.3. 16S rRNA Gene Data Processing

PCR primers were removed and sequence data screened for quality using Trimmomatic (version 0.32 (Bolger et al., 2014 [25])) to remove sequences with an average phred score < 35 and ambiguous bases (N’s). Paired read assembly was performed using FLASH (version 1.2.11, Magoc and Salzberg, 2011 [26]). UChime was used to eliminate chimeric amplicons (Edgar et al., 2011 [27]). Using BMTagger (version: 3.101, (BMTagger, RRID: SCR 014619), possible host-derived sequences were removed from reads by comparing them to the mouse or human reference genome for mice or human datasets, respectively. Assembled, filtered amplicons were binned into operational taxonomic units (OTU) using USEARCH V11 (Edgar, 2010 [28]) at a 97% identity threshold. The Ribosomal Database Project (RDP 2020) classifier was used for the taxonomic assignment of OTU sequences (Wang et al., 2007 [29] and Cole et al., 2014 [30]).

### 4.4. Statistical Analysis and Graphical Display

Statistical analysis and data visualization were performed using R (version 4.* (R Core Team, 2021 [31]) and RStudio (version 2022.02.3 Prairie Trillium): Phyloseq (McMurdie et al., 2013 [32]), ggplot2 (Wickham, 2016 [33]), vegan (Oksanen et al., 2022 [34]), DESeq2 (Love et al., 2014 [35]), stats (R Core Team, 2022 [36]), ggpubr (Kassambara, 2020 [37]), plyr (Wickham, 2011 [38]), devtools (Wickham et al., 2021 [39]), microbiomeSeq (Ssekagiri, 2022 [40]), tidyverse (Wickham et al., 2019 [41]), reshape (Wickham, 2007 [42]), and janitor (Firke, 2021 [43]).

### 4.5. Quality Assurance

To ensure data accuracy, negative control samples were analyzed to identify potential contaminants. A minimal number of unwanted microbial sequences (OTUs) were detected in these controls, with a maximum of 16 sequence reads detected in any single OTU. To eliminate the potential impact of these contaminants, all sequence reads with fewer than 30 occurrences were removed from the entire dataset, thereby improving data quality and reliability.

### 4.6. Statistical Comparison Between the Rapid and HMP Methods

The relative abundances of microbial taxa were summarized at the genus and species levels and represented as percentage values, both with and without log transformation. Alpha diversity was examined with the Pielou’s evenness (Pielou, 1966 [44]), Moore’s richness (Moore 2013 [45]), Shannon’s diversity (Shannon, 1948 [46]), and Chao1 diversity (Chao et al., 1992 [47]) methods. Beta diversity was explored using Bray–Curtis dissimilarity (Bray et al., 1957 [48]).

Wilcoxon tests (Wilcoxon, 1945 [49]) and Student’s *t*-tests (Student, 1908 [50]) were employed for pairwise comparisons. Principal coordinates analysis (PCoA) was based on a Bray–Curtis distance. Random Forest (Breiman, 2001 [51]) was implemented to identify genera that were most informative for distinguishing between the two methods.

To generate a consensus of bacterial taxa that consistently discriminated between the Rapid and HMP methods across different statistical approaches, an overall significance ranking score was calculated based on nine different methods (percentage prevalence difference, percentage abundance difference, Bray–Curtis distance, Wilcoxon test *p*-value, overall mean abundance percentage, log2(fold change), fold change *p*-value, Random Forest mean decrease accuracy, and Random Forest means decrease Gini). For each of these nine values, distinct ranking lists were issued, and the ultimate ranking was determined by taking the ninth root of the product of all ranking scores.

### 4.7. Understanding Primer Bias and Optimization

The HMP protocol followed in this study made use of a single forward/reverse primer set to cover the V1–V3 variable region of the 16S rRNA gene (V1: AGAGTTTGATCCTGGCTCAG and V3: GTGCCAGCAGCCGCGGTAA). By contrast, the Rapid protocol made use of degenerate base positions within these forward and reverse sequences to generate eleven possible V1 primers and seven possible V3 primers. To understand the possible impact of increased primer diversity on results observed when comparing these two methods, forward and reverse primers were matched against 12,990 curated sequences present in the Intus Biosciences’ proprietorial Athena database. Instances where a 100% match to a reference sequence could not be found for one or more forward/reverse primers were recorded as a mismatch for the taxon from which the sequence originated.

## Figures and Tables

**Table 1:** The top 20 genera differentiating the Rapid and HMP methods were identified based on a combined significance ranking. These genera are presented for mouse fecal and human stool microbiome datasets, respectively.

	Ranking		Matched Primers with Each Method Based on Athena Database				Number of Detected OTUs		Prevalence Percentage	
			V1 Forward Primer		V3 Reverse Primer					
	Final Rank	Significant Genera	Rapid	HMP	Rapid	HMP	Rapid	HMP	Rapid	HMP
Mouse	1	*Lactobacillus*	1, 9	Yes	1	Yes	2	1	93	21
	2	*Ihubacter*	Doesn’t exist in Athena database				3	1	99	7
	3	*unclassified_Eggerthellaceae*	1	Yes	1	Yes	7	0	61	0
	4	*unclassified_Lachnospiraceae*	1, 7	Yes	1	Yes	216	172	99	99
	5	*Duncaniella*	Doesn’t exist in Athena database				23	21	91	89
	6	*Adlercreutzia*	1	Yes	1	Yes	7	1	73	2
	7	*Longibaculum*	Doesn’t exist in Athena database				1	1	49	1
	8	*unclassified_Clostridiales*	1, 6, 7, 9	Yes	1	Yes	27	18	100	86
	9	*unclassified_Bacteroidales*	1, 9	Yes	1	Yes	13	9	100	100
	10	*Clostridium XVIII*	Doesn’t exist in Athena database				1	1	92	35
	11	*unclassified_Firmicutes*	1, 6, 7, 8, 9	Yes	1, 6, 7	Yes	16	6	65	1
	12	*unclassified_Ruminococcaceae*	1, 6	Yes	1	Yes	35	28	93	23
	13	*Intestinimonas*	1	Yes	1	Yes	5	5	97	92
	14	*unclassified_Erysipelotrichaceae*	1	Yes	1	Yes	7	2	70	89
	15	*Schaedlerella*	Doesn’t exist in Athena database				1	1	57	1
	16	*Lachnospiracea_incertae_sedis*	Doesn’t exist in Athena database				1	1	49	5
	17	*Acutalibacter*	1	Yes	1	Yes	3	2	46	1
	18	*unclassified_Muribaculaceae*	1	Yes	1	Yes	16	11	34	5
	19	*Ruminococcus*	1	Yes	1	Yes	2	0	33	0
	20	*Turicibacter*	1	Yes	1	Yes	1	1	35	35
Human	1	*Bacteroides*	1, 9	Yes	1, 6	Yes	7	12	70	95
	2	*Faecalibacterium*	1	Yes	1	Yes	5	5	95	85
	3	*Phocaeicola*	1	Yes	1	Yes	6	8	55	75
	4	*Blautia*	1	Yes	1	Yes	8	4	100	30
	5	*Anaerobutyricum*	Doesn’t exist in Athena database				3	2	90	10
	6	*Ruminococcus*	1	Yes	1	Yes	2	2	60	20
	7	*unclassified_Ruminococcaceae*	1, 6	Yes	1	Yes	26	21	95	95
	8	*Dorea*	1	Yes	1	Yes	1	1	85	55
	9	*Coprococcus*	1	Yes	1	Yes	3	1	55	5
	10	*Collinsella*	7	No Match	1	Yes	1	2	65	20
	11	*Romboutsia*	Doesn’t exist in Athena database				1	1	65	50
	12	*Parabacteroides*	1	Yes	1	Yes	3	5	25	60
	13	*Anaerostipes*	1	Yes	1	Yes	2	2	70	25
	14	*unclassified_Lachnospiraceae*	1, 7	Yes	1	Yes	22	17	80	50
	15	*unclassified_Clostridiales*	1, 6, 7, 9	Yes	1	Yes	12	11	55	55
	16	*Prevotella*	1	Yes	1	Yes	4	6	40	55
	17	*Mediterraneibacter*	Doesn’t exist in Athena database				1	0	35	0
	18	*Alistipes*	1	Yes	1	Yes	1	4	5	45
	19	*Faecalibacillus*	Doesn’t exist in Athena database				2	2	75	65
	20	*Roseburia*	1	Yes	1	Yes	3	2	60	40

## Data Availability

The original contributions presented in this study are included in the article/Supplementary Material. Further inquiries can be directed to the corresponding author.

## Supplementary Materials

The following supporting information can be downloaded at: https://www.mdpi.com/article/10.3390/ijms26031180/s1.
