# Supplementary material for: Comparison of Lysis and Amplification Methodologies for Optimal 16S rRNA Gene Profiling for Human and Mouse Microbiome Studies
Source: Int J Mol Sci. 2025 Jan 29;26(3):1180. doi: 10.3390/ijms26031180 (PMC11818825; doi:10.3390/ijms26031180)
Supplement: Supplementary file 1 [file ijms-26-01180-s001.zip › ijms-3368218 supplementary ma.pdf]

Comparison of Lysis and Amplification Methodologies for Optimal 16S rRNA Gene Profiling for Human and Mouse Microbiome Studies

Supplementary Figures and Tables

Table S1. The number of samples prepared for each strain using HMP and Rapid methods.

Strain		# of samples preped by		
		HMP	Rapid	Total
Inbred Strain	C57BL/6J	2	8	10
	A/J	2	8	10
Chromosome Substitution Strain	C57BL/6J-Chr01<A/J>/NaJ	4	6	10
	C57BL/6J-Chr02<A/J>/NaJ	4	6	10
	C57BL/6J-Chr03<A/J>/NaJ	4	6	10
	C57BL/6J-Chr04<A/J>/NaJ	4	6	10
	C57BL/6J-Chr05<A/J>/NaJ	4	6	10
	C57BL/6J-Chr06<A/J>/NaJ	4	6	10
	C57BL/6J-Chr07<A/J>/NaJ	4	6	10
	C57BL/6J-Chr08<A/J>/NaJ	4	6	10
	C57BL/6J-Chr09<A/J>/NaJ	4	6	10
	C57BL/6J-Chr10<A/J>/NaJ	4	6	10
	C57BL/6J-Chr11<A/J>/NaJ	4	6	10
	C57BL/6J-Chr12<A/J>/NaJ	4	6	10
	C57BL/6J-Chr13<A/J>/NaJ	4	6	10
	C57BL/6J-Chr14<A/J>/NaJ	4	6	10
	C57BL/6J-Chr15<A/J>/NaJ	4	6	10
	C57BL/6J-Chr16<A/J>/NaJ	4	6	10
	C57BL/6J-Chr17<A/J>/NaJ	4	6	10
	C57BL/6J-Chr18<A/J>/NaJ	4	6	10
	C57BL/6J-Chr19<A/J>/NaJ	4	6	10
	C57BL/6J-ChrX<A/J>/NaJ	4	6	10
Total # of samples		84	136	220

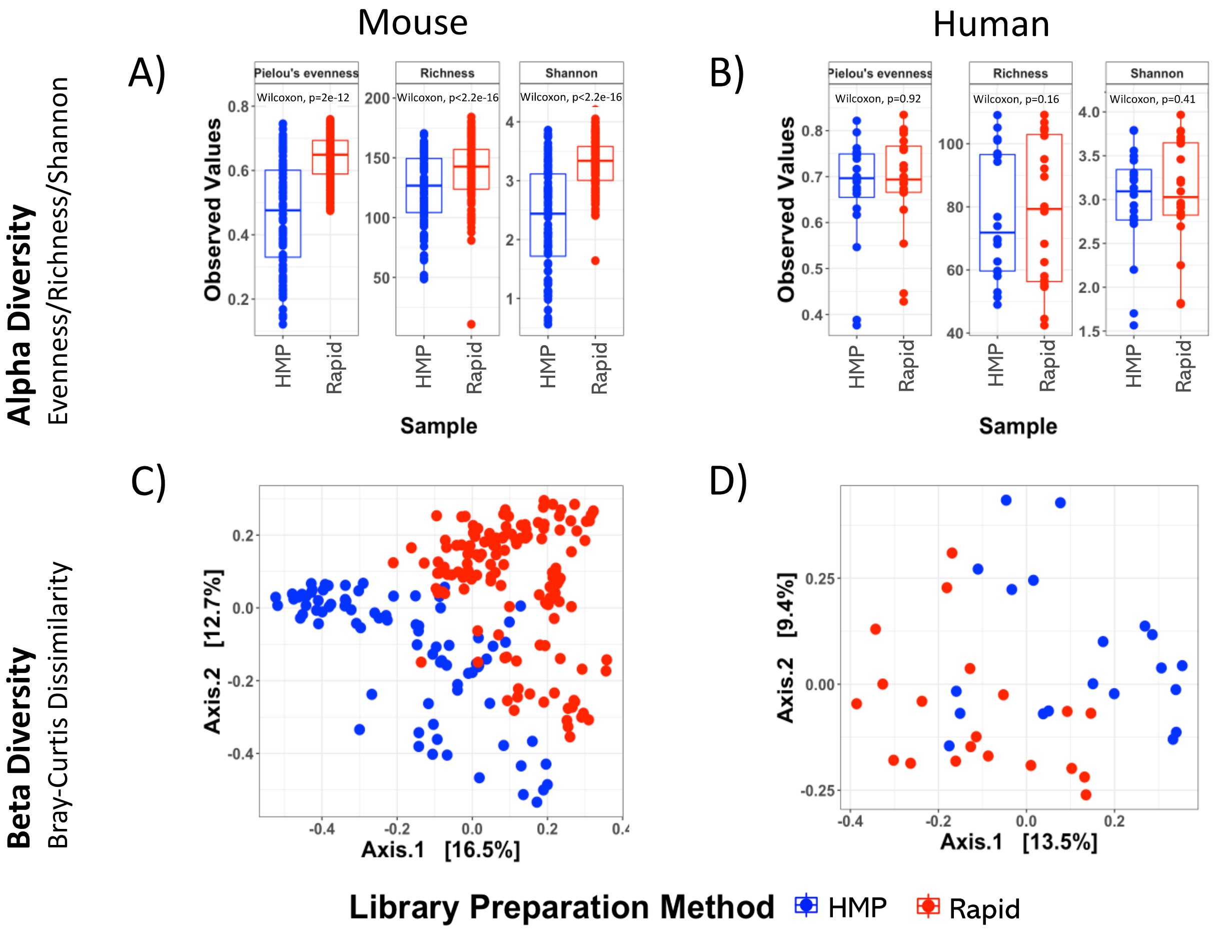

Figure S1. Method Effects on Microbiome Diversity and Sample Similarity: A Comparison of HMP and Rapid 16S rDNA Extraction. **A, B)** Alpha diversity calculated at the OTU level for mouse fecal and human stool samples extracted using either the HMP or Rapid technique. Each data point represents the alpha diversity measurement for one sample. Boxes show the distribution of data points within each group with a line showing the mean value. Wilcoxon p-values are shown on each panel. **C, D)** PCoA plots to visually represent difference in microbiome community composition between samples. Each data point represents one sample. The percentage values on each axis represent the proportion of variance explained by each of the first two principal coordinate axes.

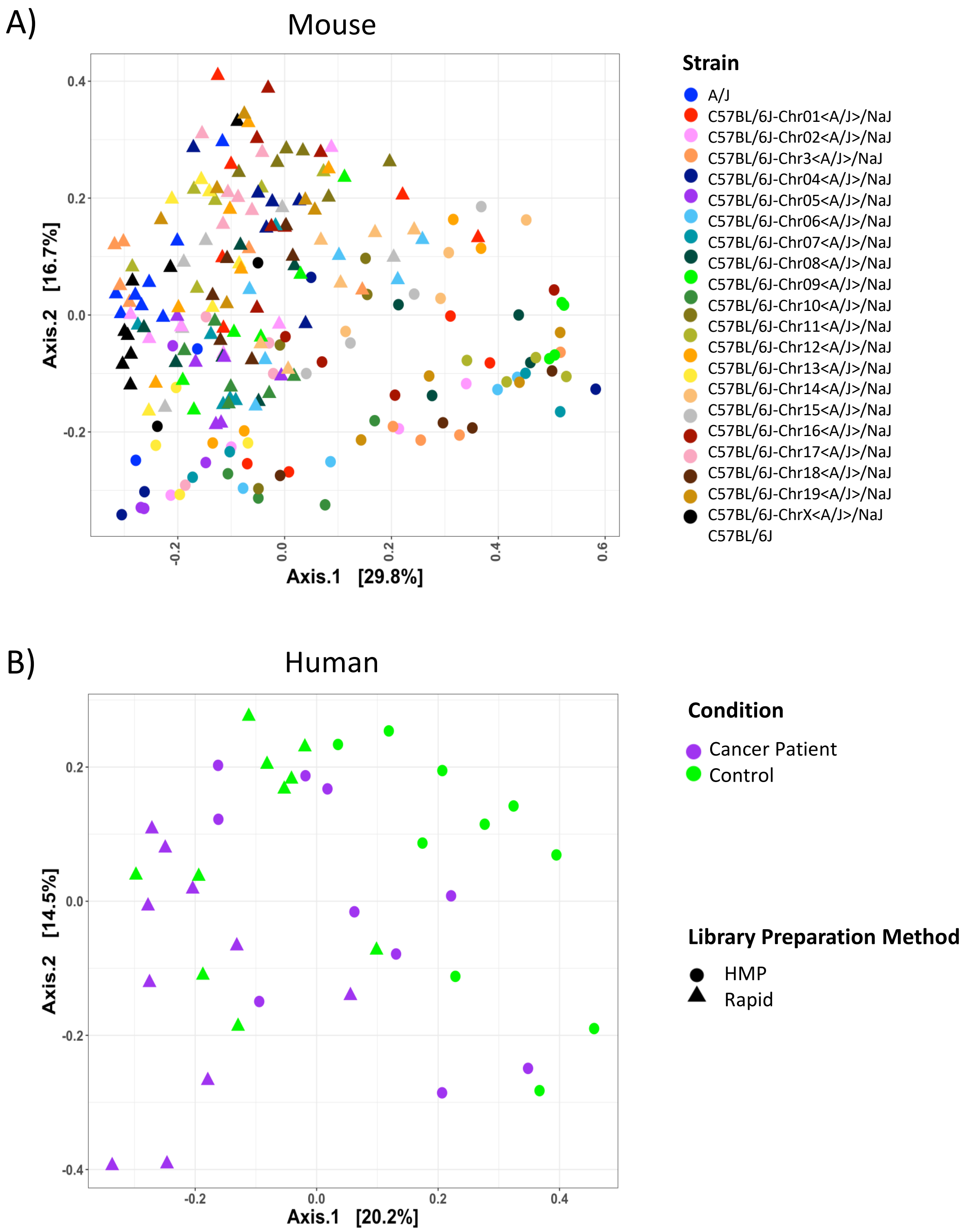

Figure S2. Bray-Curtis Dissimilarity Between Samples A, **B)** PCoA plots to visually represent difference in microbiome community composition between samples.
